# Multifunctional and bioinspired titanium surface with multilayer nanofilms for novel dental implant applications

**DOI:** 10.3389/fchem.2024.1426865

**Published:** 2024-07-05

**Authors:** Caiyun Wang, Ran Lu, Xu Cao, Yanting Mu, Su Chen

**Affiliations:** Laboratory of Biomaterials and Biomechanics, Beijing Key Laboratory of Tooth Regeneration and Function Reconstruction, Beijing Stomatological Hospital, Capital Medical University, Beijing, China

**Keywords:** titanium implants, nanofilms, bioinspired design, sustained-release carrier, multifunctional surface

## Abstract

**Introduction:** Smart multifunctional surfaces targeting intricate biological events or versatile therapeutic strategies are imminent to achieve long-term transmucosal implant success.

**Methods:** This study used dopamine (DA), graphene oxide (GO), and type IV collagen (COL-IV) to construct multilayer nanofilms (DGC_n_) based on their universal adhesive and biomimetic properties to design a versatile and bioactive titanium implant. The characterization of DGC_n_ on different titanium surfaces was performed, and its loading capacity, release profile, *in situ* gene delivery, and *in vitro* biological properties were preliminarily evaluated.

**Results:** Our results demonstrate that hydrogenated TiO_2_ nanotubes (H) provide a better platform for the DGC_n_ coating than machined Ti and air-TiO_2_ nanotubes. The H-DGC_10_ displayed the most stable surface with excellent loading capacity, sustained-release profile, and *in situ* gene transfection efficiency; this could be due to the high specific surface area of H and GO, as well as the functional groups in H, DA, and GO. Moreover, the H-DGC_10_ exhibited good biocompatibility for human oral epithelial cells and promoted the expression of integrin β4 and laminin 332, both being hemidesmosome-related proteins.

**Discussion:** Our findings suggest that H-DGC_n_ can be designed as a smart multifunctional interface for titanium implants to achieve long-term transmucosal implant success and aid in versatile therapeutic strategies.

## 1 Introduction

The long-term success of dental implants is not only limited to stable osseointegration but also to the sealing effect of transmucosal soft tissues. Firm soft-tissue integration can resist mechanical stress, microbial invasion, and marginal bone resorption (Saso and Lee, 2018; Ahamed et al., 2021; Kunrath and Gerhardt, 2023). However, the implant–soft-tissue interface can exhibit inferior sealing functionality compared with natural teeth, leading to various biological complications such as peri-implant mucositis and peri-implantitis (Iacov-Craitoiu and Craitoiu, 2020; Weinstein et al., 2020). Titanium—the preferred biomaterial for dental implants—has been widely investigated to facilitate osseointegration. Currently, well-modified titanium surfaces for soft-tissue sealing are at the forefront of implant research.

Nanostructured, biomimetic, and antibacterial surfaces have been demonstrated to modulate soft-tissue integration and reduce biofilm formation (Guo et al., 2021; Areid et al., 2024; Shrivas et al., 2024). However, most modified surfaces have been developed with a single function for a specific objective, and may not cope with intricate peri-implant microenvironments. In practice, versatile surfaces are required. Mathur *et al.* (Mathur et al., 2022) fabricated a gelatin electrospun mat scaffold embedded with silver nanoparticles on a titanium alloy surface to improve fibroblast adhesion, differentiation, and antimicrobial activity. Boda *et al.* (Boda and Aparicio, 2022) introduced adhesive peptides and anti-inflammatory biomolecules into a dual-function titanium coating to reduce the inflammatory response and improve soft-tissue adhesion. Another study fabricated multilayer alginate/chlorhexidine coatings on titanium surfaces and demonstrated that the modified coatings inhibited plaque biofilm formation and decreased inflammation *in vivo* (Wu et al., 2023). Importantly, the coatings led to the adhesion and proliferation of fibroblasts, even in a bacterial environment. However, these methods are often designed to load a specific substance for a specific biological effect, which is not conducive to their general application or popularization. Consequently, a smart multifunctional surface with bioactive, immunomodulatory, and antibacterial properties that targets complicated and diverse biological events and versatile therapeutic strategies (such as bioactive molecule/drug/gene delivery during soft-tissue integration), is urgently needed.

Dopamine (DA) is a biomimetic substance derived from mussel adhesive proteins that can produce polydopamine (PDA) with more functional groups via self-polymerization under alkaline conditions. PDA is widely used to construct multifunctional bioengineering materials owing to its excellent interfacial interactivity, bioactivity, and antioxidant capacity (Liu et al., 2016; Ball, 2018; Xie et al., 2021). Qin *et al.* (Qin et al., 2022) used PDA to immobilize BMP-2 gene encapsulated in aminated poly (lactic-co-glycolic acid) microspheres on polyetheretherketone. They verified that the PDA coating enhanced biological activity and that gene delivery effectively improved osteogenic differentiation. Another study developed a multifunctional nanosystem with macrophage cell membrane-camouflaged oseltamivir-PDA nanoparticles (Liu et al., 2023b). PDA nanoparticles have been shown to suppress inflammatory storms by removing reactive oxygen species, leading to controlled drug release. Based on these properties, DA was employed as a biomimetic substance and an adhesive interface between titanium surfaces and other layers in this study.

Graphene oxide (GO) has recently emerged as a promising material for biological applications. Sharma *et al.* (Sharma et al., 2022) coated a polylactic acid scaffold with PDA-reduced GO, and the designed coating exhibited antioxidant and antimicrobial properties, as well as pro-angiogenic and osteoinductive functionality. Kutwin *et al.* (Kutwin et al., 2021) used graphene-based complexes as miRNA vectors to support anticancer therapy. In another study, GO was coated on collagen membranes to enhance their biocompatibility (Radunovic et al., 2017), with the GO-modified membranes inducing stem cell differentiation and reducing inflammation. Additionally, GO possesses a high specific surface area and abundant active groups (such as hydroxyl, carboxyl, and epoxy groups), which induce biochemical and bioconjugation reactions (Tiwari et al., 2020). In this study, we sought to take advantage of DA and GO to develop a flexible and versatile titanium coating to achieve excellent multifunctional integration. Impressively, GO has been widely applied in the regeneration, antimicrobial, anti-inflammatory, and gene/drug delivery fields (Hoseini-Ghahfarokhi et al., 2020; Raslan et al., 2020; Ramirez and Osendi, 2022; Inchingolo et al., 2023). Recently, bioactive coatings incorporating GO have been constructed using the layer-by-layer (LBL) technology. A previous study employed LBL to fabricate a multilayer coating on magnesium alloy surface, utilizing chitosan-functionalized GO (GOCS) and heparin (Hep). This coating not only improved corrosion resistance but also enhanced biocompatibility with endothelial cells (Gao et al., 2020). In another study, a bioinspired PDA/GO/collagen coating was constructed using LBL, serving as a multifunctional carrier for bioactive components (Xu et al., 2021). Furthermore, You *et al.* (You et al., 2024) developedε-poly-L-lysine (PLL)/GO self-assembly multilayers, demonstrating that 20 layers of PLL/GO exhibited remarkable antibacterial properties without any biological toxicity.

Biomimetic natural extracellular matrix (ECM) components provide a comparable microenvironment for cell communication, mechanotransduction, structural integrity, and signal regulation in biomedical engineering (Tamayo-Angorrilla et al., 2022; Liu et al., 2023a; Zhu et al., 2023). Type Ⅳ collagen (COL-Ⅳ)—a pivotal component of the basement membrane (BM), a specialized thin matrix of ECM that is critical for peri-implant soft tissue sealing—can integrate with laminin polymeric networks for epithelial cell anchoring. Coelho-Sampaio *et al.* (Coelho-Sampaio et al., 2020) fabricated a flat BM-like network by assembling COL-Ⅳ with poly-laminin, which was shown to be conducive to the formation of stratified cell layers, organized F-actin, and tight junctions. Another study designed a dense COL-Ⅳ and/or laminin layer on type Ⅰ collagen film to mimic the Descemet’s membrane (Palchesko et al., 2016). Moreover, Zeng *et al.* (Zeng et al., 2020) developed a multilayer COL-Ⅳ/laminin nanofilm, and demonstrated that the mimetic BM improved cell adhesion and spreading, while inhibiting cell migration. Consequently, constructing a biomimetic BM structure is potentially beneficial for sealing transmucosal soft tissue.

In this study, we designed a biomimetic, flexible, and versatile system as a template to target diverse biological events and develop therapeutic strategies to promote peri-implant soft-tissue sealing. Accordingly, multilayer DA/GO/COL-IV nanofilms were coated onto different titanium surfaces using a LBL technique. The surface characteristics, loading/delivery capabilities, and inherent bioactivities of the modified surfaces were evaluated.

## 2 Materials and methods

### 2.1 Materials

Machined titanium specimens (99.99%) were purchased from Cuibolin Nonferrous Metal Industry Co., Ltd. (Beijing, China). Acetone, ethyl alcohol, ethylene glycol, and ammonium fluoride were purchased from Sinopharm Chemical Reagent Co., Ltd. (Shanghai, China). XFNANO (Nanjing, China) provided us with graphene oxide (GO) dispersion water (2 mg/mL, lateral size 50–200 nm). Dopamine (DA) hydrochloride, type Ⅳ collagen (COL-Ⅳ), bovine serum albumin (BSA), and QuantiPro BCA Assay Kit were purchased from Sigma-Aldrich (Merck, Darmstadt, Germany). Sirius Red Total Collagen Detection Kit was supplied by Chondrex, Inc. (Woodinville, WA, United States). Recombinant adenoviral vectors expressing mCherry (Ad-mCherry) were constructed by HANBIO (Shanghai, China). Human oral epithelial cells (HOECs) were purchased from Wuhan Pricella Biotechnology Co., Ltd. (Wuhan, China). Anti-adenovirus type 5 antibody, rabbit antibody targeting integrin β4, and DyLight 488-conjugated anti-rabbit IgG were supplied by Abcam (Cambridge, United Kingdom). Paraformaldehyde (4%), Triton X-100, goat serum, 3-(4, 5-Dimethyl-2-thiazolyl)-2, 5-diphenyl-2H-tetrazolium bromide (MTT), and Calcein/PI Live/Dead Assay Kit were purchased from Beyotime Biotechnology (Beijing, China). 4′,6-Diamidino-2-phenylindole (DAPI) was purchased from ZSGB-BIO (Beijing, China). Dulbecco’s Modified Eagle Medium (DMEM), fetal bovine serum (FBS), and penicillin/streptomycin were obtained from Gibco (Thermo Fisher Scientific Inc., United States). TRIzol Reagent kit was purchased from Invitrogen (Carlsbad, CA, United States). PrimeScript RT Reagent kit was purchased from TaKaRa (Shiga, Japan). RT-PCR reagent was obtained from CWBio (Beijing, China). The primer sequences for the target genes were constructed by Shenggong (Shanghai, China).

### 2.2 Pretreatment of titanium specimens

Machined titanium specimens (10 mm × 10 mm × 0.2 mm) were used in this study. Ultrasonic cleaning was performed by rinsing the specimens with acetone, ethyl alcohol, and distilled water for 5 min, respectively. Anodic oxide specimens were then prepared by means of an electrochemical method (at 50 V for 15 min) using Ti as the anode and ethylene glycol (0.5 wt% ammonium fluoride and 10 vol% deionized water) as the electrolyte. After annealing at 500°C for 2 h in air, the specimens were labeled as air-TiO_2_ nanotubes (A group). Subsequently, hydrogenated TiO_2_ nanotubes (H group) were prepared using a thermal hydrogenation technique under a hydrogen atmosphere (0.95 × 10^5^ Pa, 500°C, and 4 h). Machined titanium (T) specimens were used as controls.

### 2.3 Preparation of multilayer DA/GO/COL-Ⅳ (DGC) nanofilms

The layer-by-layer (LBL) self-assembly method was employed to fabricate multilayer DGC nanofilms on the surfaces of the T, A, and H groups. First, the specimens were dipped in a DA/Tris solution (2.0 mg/mL, pH = 8.5) at 25°C. After 5 min, the specimens were washed thrice and immersed in a GO suspension (0.5 mg/mL) for another 5 min. The specimens were then assembled with COL-IV in a COL-Ⅳ/acetic acid buffer solution (50 μg/mL, pH = 4.5) for 5 min. Following each immersion, the specimens were cleaned three times with deionized water to remove unbound components. By repeating this process 5, 10, and 20 times, multilayer DGC nanofilms (DGC_n_, where n denotes the number of DGC layers) were fabricated on the surfaces of the T, A, and H groups (denoted as T-DGC_n_, A-DGC_n_, and H-DGC_n_, respectively). All specimens intended for *in vitro* experiments were disinfected using an ultraviolet device for 20 min on both sides.

### 2.4 Characterization of specimens

Scanning electron microscopy (SEM, SU8010, Hitachi, Ltd., Tokyo, Japan) was employed to observe the surface morphology of the specimens. To determine the thickness of the nanofilms, the specimens were embedded in resin, and cross-sectional slices were obtained for SEM analysis. The elemental distribution on the specimens was analyzed through energy dispersive spectroscopy (EDS) equipped with SU8010 SEM. The surface roughness was analyzed using atomic force microscopy (AFM; Dimension ICON, Bruker, Germany). The contact angles (CAs) of the specimens were measured using an optical system (OCA20; Data Physics Instruments, Esslingen, Germany). The bonding strengths of the nanofilms were assessed using a nanoscratch test (TI 980, Bruker). Surface elemental composition and chemical state analyses were performed using X-ray photoelectron spectroscopy (XPS; ESCALAB Xi+, Thermo Scientific, United States).

### 2.5 Evaluation of COL-Ⅳ encapsulation

To evaluate the encapsulation capacities of the T-DGC_n_, A-DGC_n_, and H-DGC_n_, quantitative amounts of COL-IV were evaluated. Briefly, the T-DGC_n_, A-DGC_n_, and H-DGC_n_ specimens were placed in a 24-well plate, before being repeatedly scraped with a pipette tip in 0.05 M acetic acid (350 μL/well) to collect the encapsulated nanofilms. Subsequently, ultrasonic treatment was performed for 10 min. The collected samples were analyzed using the Sirius Red Total Collagen Detection Kit according to the manufacturer’s instructions. The optical density (OD) was measured at 520 nm using a microplate spectrophotometer (SpectraMax Paradigm, Molecular Devices, CA, United States). The amount of COL-IV encapsulated in the DGC multilayers was calculated using regression analysis based on the standard curve. The T-DGC_10_, A-DGC_10_, and H-DGC_10_ specimens were stained with Sirius Red and observed under a stereoscopic microscope (Leica, Hamburg, Germany). The optical images were captured using a digital camera.

### 2.6 Evaluation of protein release

To evaluate the release profile of the DGC nanofilms, BSA was used as a model bioactive compound loaded onto the A-DGC_10_ and H-DGC_10_. In brief, specimens were immersed in a BSA solution (1 mg/mL) for 5 min between GO and COL-Ⅳ assembly during fabrication of each DGC layer. After 10 repeated cycles, the A and H specimens loaded with BSA-encapsulated DGC multilayers were denoted as A-DGBC_10_ and H-DGBC_10_, respectively. The A-DGBC_10_ and H-DGBC_10_ specimens were then dipped into 350 μL phosphate buffered saline (PBS, pH = 7.4), followed by incubation at 37°C and 98% relative humidity. After 2, 4, 6, 8, 12, 24, 48, 72, 120, 168, 240, and 336 h of incubation, 100 μL PBS containing the released protein (COL-Ⅳ and BSA) was removed at each time point and 100 μL fresh PBS was added. The collected protein samples were quantitatively detected using a QuantiPro BCA Assay Kit according to manufacturer’s instructions. After 168 and 336 h of release, the A-DGBC_10_ and H-DGBC_10_ specimens were removed and washed three times with deionized water. The surface morphology was observed using SEM (Hitachi Ltd., Japan).

### 2.7 Transfection efficiency

To evaluate the capability of the DGC nanofilms as an *in situ* gene delivery system, the transfection efficiency of cells on different specimens was evaluated. Ad-mCherry was used as gene vector models. An anti-adenovirus-functionalized surface, as described previously (Lin et al., 2010) was used as the positive control. In brief, the H-DGC_10_ samples were incubated with anti-adenovirus type 5 antibody (Ab; 1:1,000) overnight at 4°C. After three washes with PBS, the samples were labeled as H-DGC_10_-Ab. Subsequently, the H, H-DGC_10_, and H-DGC_10_-Ab were incubated with Ad-mCherry (0.1, 0.5, 1.0, and 2.0 × 10^8^ PFU/mL) in 24-well plates at 37°C for 4 h. Following three cycles of washing to remove the unbonded Ad-mCherry, HOECs were seeded on the specimens at a density of 1 × 10^5^ cells/well. After being transfected for 3 days, the cells were fixed with 4% paraformaldehyde and stained with DAPI. The samples were observed under a fluorescence microscope (Olympus). The percentage of mCherry-positive cells in three randomly selected microscopic fields was calculated using ImageJ software.

### 2.8 Evaluation of biological effects on HOECs

#### 2.8.1 Cell culture

The HOECs were cultured in a complete DMEM supplemented with 10% FBS and 1% penicillin/streptomycin at 37°C in a 5% CO_2_ atmosphere.

#### 2.8.2 Cell viability and proliferation

The effects of the T, A, H, T-DGC_10_, A-DGC_10_, and H-DGC_10_ on cell viability were evaluated using live/dead cell staining and the MTT assay. The HOECs were seeded onto the specimens at a density of 3 × 10^5^ cells/well in 24-well plates. After culturing for 24 h, the cells were stained using a Calcein/PI Live/Dead Assay Kit according to the manufacturer’s instructions. The samples were observed under a fluorescence microscope (BX51; Olympus, Tokyo, Japan), and representative images were captured. The quantitative living cell ratio (%) was analyzed using ImageJ software.

To assess cell proliferation on the T, A, H, T-DGC_10_, A-DGC_10_, and H-DGC_10_ surfaces, the HOECs were seeded in 24-well plates (3 × 10^5^ cells/well). After incubation for 1, 3, and 5 days, the cell proliferation was evaluated using MTT. Briefly, at each time point, the medium was replaced by 350 µL complete medium containing 10% 5 mg/mL MTT solution in each well. The MTT was reduced to formazan pigment by living cells after being incubated at 37°C for 4 h. Subsequently, formazan pigment was dissolved using 350 µL dimethyl sulfoxide, and the solution was transferred to 96-well plates (100 µL/well). The OD values were measured using a microplate spectrophotometer (SpectraMax Paradigm, United States) at 570 nm.

#### 2.8.3 Immunofluorescence

Integrin β4, a hemidesmosome-related protein, was determined using immunofluorescence assay. The HOECs were cultured on different surfaces at a density of 5 × 104 cells/well in 24-well plates for 48 h. After three washes with PBS, the cells were fixed with 4% paraformaldehyde at room temperature for 15 min, followed by permeabilization with 0.1% Triton X-100 for 10 min. Subsequently, the cells were blocked with 10% goat serum for 30 min and incubated with a specific primary rabbit antibody targeting integrin β4 (1:250 dilution) overnight at 4°C. After three washes, the cells were incubated with DyLight 488-conjugated anti-rabbit IgG (1:400 dilution) in the dark for 1 h at room temperature and with DAPI for 5 min. Fluorescent images were obtained using a fluorescence microscope equipped with a camera (Olympus, Tokyo, Japan).

#### 2.8.4 Quantitative real-time polymerase chain reaction (RT-PCR)

RT-PCR was conducted to evaluate the relative gene expression levels in HOECs on different surfaces. HOECs were seeded on T, A, H, T-DGC_10_, A-DGC_10_, and H-DGC_10_ at a density of 5 × 10^5^ cells/well in six-well plates. After 48 h of incubation, total RNA was extracted from the HOECs using a TRIzol Reagent kit. Subsequently, all samples underwent reverse transcription with a PrimeScript RT Reagent kit. The expression levels of integrin β4 (ITGB4) and laminin 332 (LAMA3) were then determined using RT-PCR reagent. To normalized the Ct values, GAPDH expression was used as an internal control. The relative gene expression was calculated using the 2^(−ΔΔCt)^ method. The primer sequences for the target genes are listed in Table 1.

**TABLE 1:** Primer pairs used in RT-PCR analysis.

Gene	Forward primers (5′ to 3′)	Reverse primers (5′ to 3′)
LAMA3	CGT​CTT​GGC​TCA​CTC​TGT​ATT	GGC​TGA​CTT​CCG​ATG​TGT​ATT​A
ITGB4	AGA​ACC​TGA​ACG​AGG​TCT​ACA	TCC​ACA​ATG​GTG​TGG​TCT​TG
GAPDH	GGA​GCG​AGA​TCC​CTC​CAA​AAT	GGC​TGT​TGT​CAT​ACT​TCT​CAT​GG

### 2.9 Statistical analysis

All quantitative values were indicated as means ± standard deviation. Statistical analyses were performed using SPSS 19.0 (International Business Machines Corporation, NY, United States) via one-way analysis of variance (ANOVA) or the Kolmogorov-Smirnov test. Statistically, *p* < 0.05 was considered significant.

## 3 Results

### 3.1 Characterization of specimens

**FIGURE 1:**
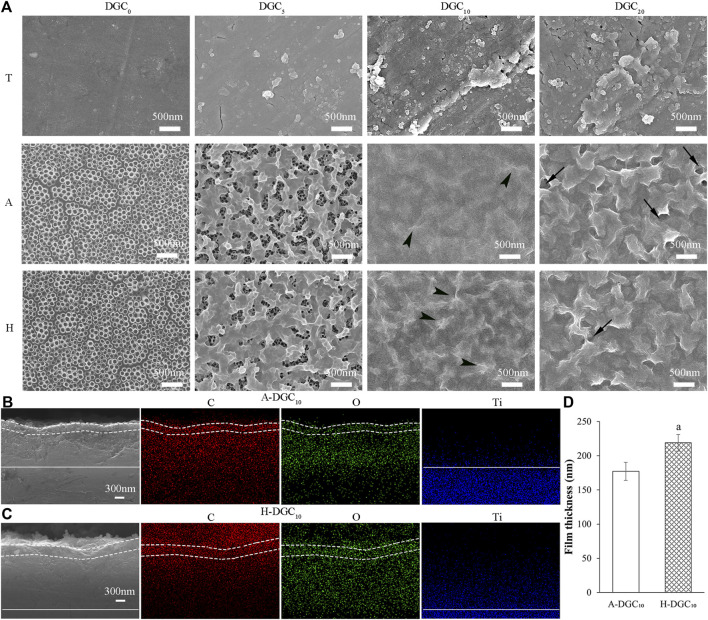
Surface topography and cross-sectional observation of different samples by SEM. **(A)** Surface topography of different samples (×30,000). Arrowheads: GO ruffles; arrows: uneven and porous defects. Cross-sectional observation and EDS mapping images of **(B)** the A-DGC_10_ group (×30,000) and **(C)** the H-DGC_10_ group (×30,000). The dashed line marks the DGC nanofilms on the surface of titanium dioxide nanotubes. The straight line marks the boundary between the titanium substrate and the nanotubes. **(D)** Thickness of nanofilms on the A-DGC_10_ and H-DGC_10_ determined from cross-sectional SEM and EDS mapping images. a, *p* < 0.05 vs the thickness of the A-DGC_10_ group.

The SEM topographies of the T-DGC_n_, A-DGC_n_, and H-DGC_n_ [where n represents 0, 5, 10, or 20 layers of dopamine/graphene oxide/type Ⅳ collagen (DA/GO/COL-Ⅳ, DGC)] are shown in Figure 1A. It is evident that group T exhibits relatively smooth surfaces, whereas groups A and H exhibit uniform nanotubes with diameters of approximately 100 nm. TiO_2_ nanotubes (TNTs) with large diameters, such as 100 nm, have been reported to exhibit increased surface energy and hydrophilicity. This enhanced property allows for greater loading capacity of proteins/drugs, ultimately leading to improved drug elution performance (Peng L et al., 2009; Kulkarni et al., 2015; Martinez-Marquez et al., 2020; Lin et al., 2021). Therefore, in this study, TNTs with a diameter of 100 nm were used as the initial platform for coating DGC nanofilms. Following assembly with five DGC layers, nanoparticles are evident in the T group, whereas nanofilm structures are formed on the nanotubes in the A and H groups (the nanofilm on the H surface covering a larger area than that on the A surface). The nanotubes in groups A and H are completely and uniformly covered with 10 and 20 DGC nanofilm layers, respectively. More GO ruffles (arrowheads) are evident in the H-DGC_10_ than in the A-DGC_10_. However, uneven and porous defects (arrows) appear on the surfaces of A-DGC_20_ and H-DGC_20_, indicating instability of the nanofilms. Additionally, the thicknesses of the DGC_10_ nanofilms were analyzed via cross-sectional observation and EDS mapping (Figures 1B,C), with the quantitative values indicating the thicknesses of the A-DGC_10_ and H-DGC_10_ to be 177 and 219 nm, respectively (Figure 1D). Unfortunately, the thickness of the T-DGC_10_ could not be reliably obtained, which may have been due to the DA/GO/COL-Ⅳ failing to form nanofilms on the T surfaces.

**FIGURE 2:**
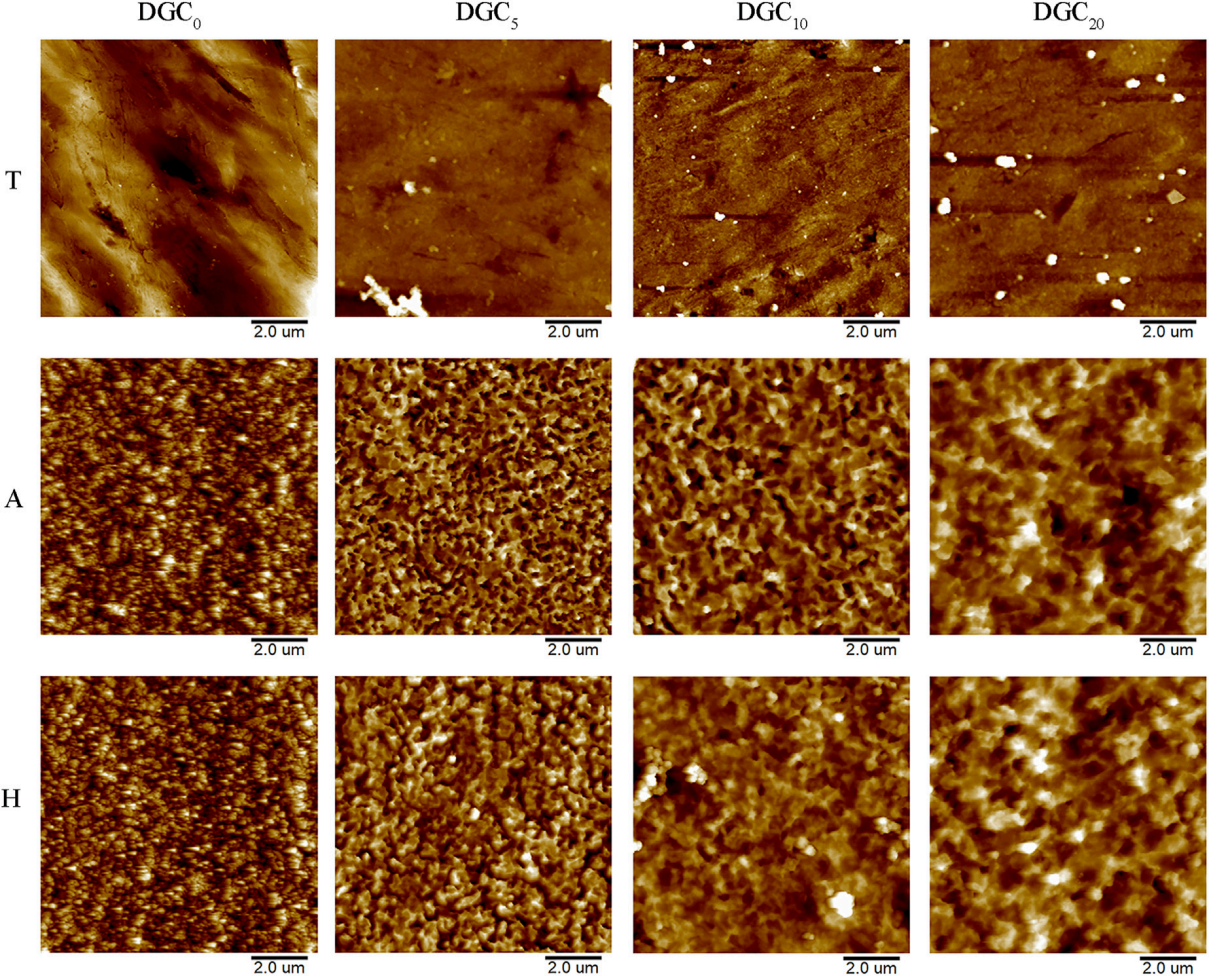
Reconstructed two-dimensional topographical images of different samples by AFM.

**FIGURE 3:**
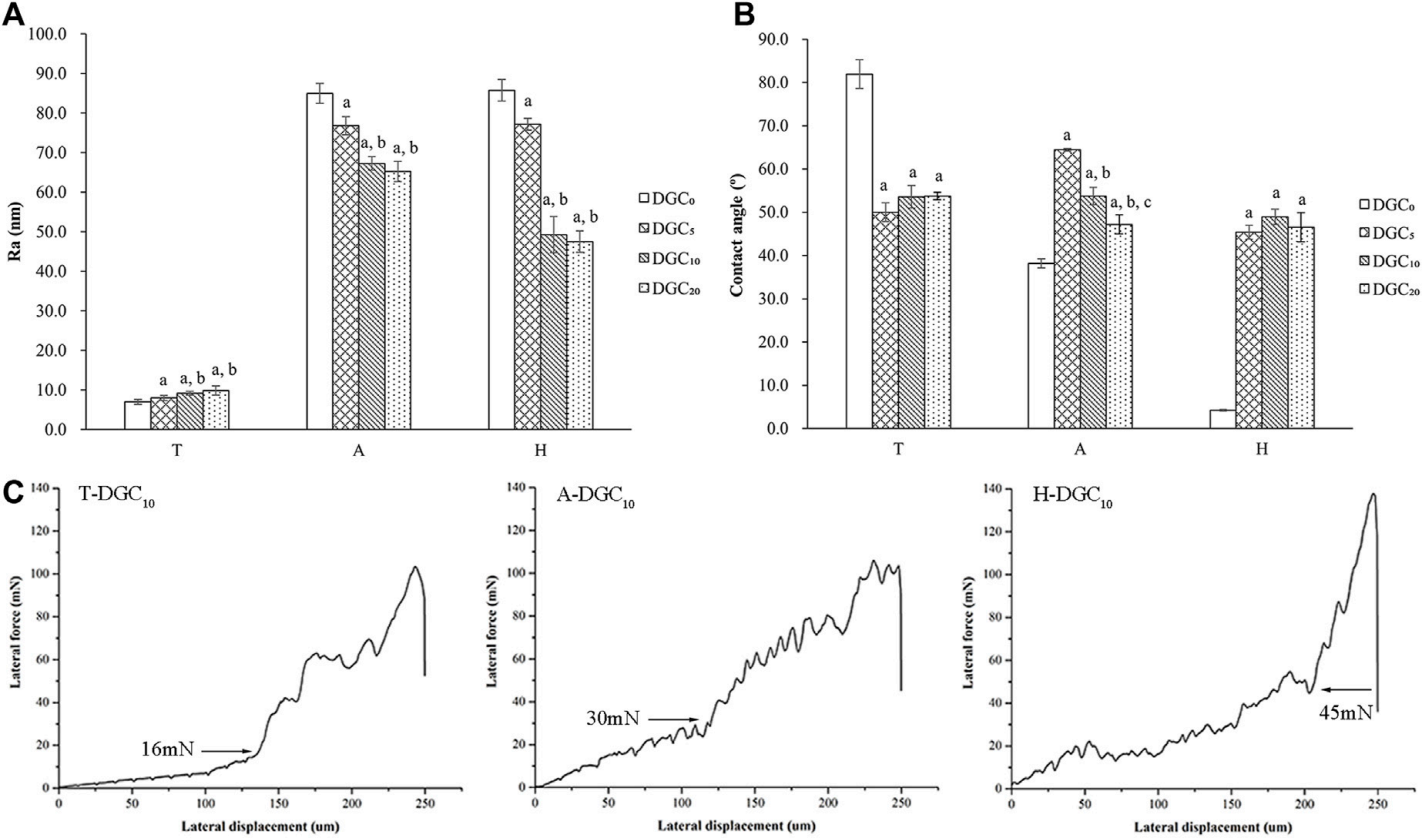
Surface physical characteristics of samples. **(A)** Surface roughness (Ra) of different surfaces determined by AFM. a, *p* < 0.05 vs the Ra value of the DGC_0_ group; b, *p* < 0.05 vs the Ra of the DGC_5_ group. **(B)** Water contact angles (CAs) of different surfaces. a, *p* < 0.05 vs the CA of the DGC_0_ group; b, *p* < 0.05 vs the CA of the DGC_5_ group; c, *p* < 0.05 vs the CA of the DGC_10_ group. **(C)** Nanoscratch test of the T-DGC_10_, A-DGC_10_, and H-DGC_10_ with critical points at 16, 30, and 45 mN, respectively.

Two-dimensional images of the surface topography obtained by AFM are shown in Figure 2, and the surface features of the different groups are consistent with those of the SEM observations. The surface roughness (Ra) of the different groups was analyzed using AFM. As shown in Figure 3A, the DGC coatings increase the Ra values of the T surfaces and decrease those of the A and H surfaces. The Ra values of the H-DGC_10_ and H-DGC_20_ are lower than that of group A. For DGC_10_ and DGC_20_ in all groups, increasing the number of layers of DGC has no effect on the surface roughness (*p* > 0.05).

Figure 3B shows the contact angle (CA) values of different samples. It is evident that the DGC coating decreases the CA values of the T surfaces and increases those of the A and H surfaces. The CA values of the T-DGC_n_ and H-DGC_n_ groups does not change significantly when the number of DGC nanofilms increases from 5 to 20 layers.

On behalf of DGC_n_ on different titanium substrates, the microtribological properties of the T-DGC_10_, A-DGC_10_, and H-DGC_10_ were evaluated using a nanoscratch test. As shown in Figure 3C, the critical loads for the T-DGC_10_, A-DGC_10_, and H-DGC_10_ are approximately 16, 30, and 45 mN, respectively.

**FIGURE 4:**
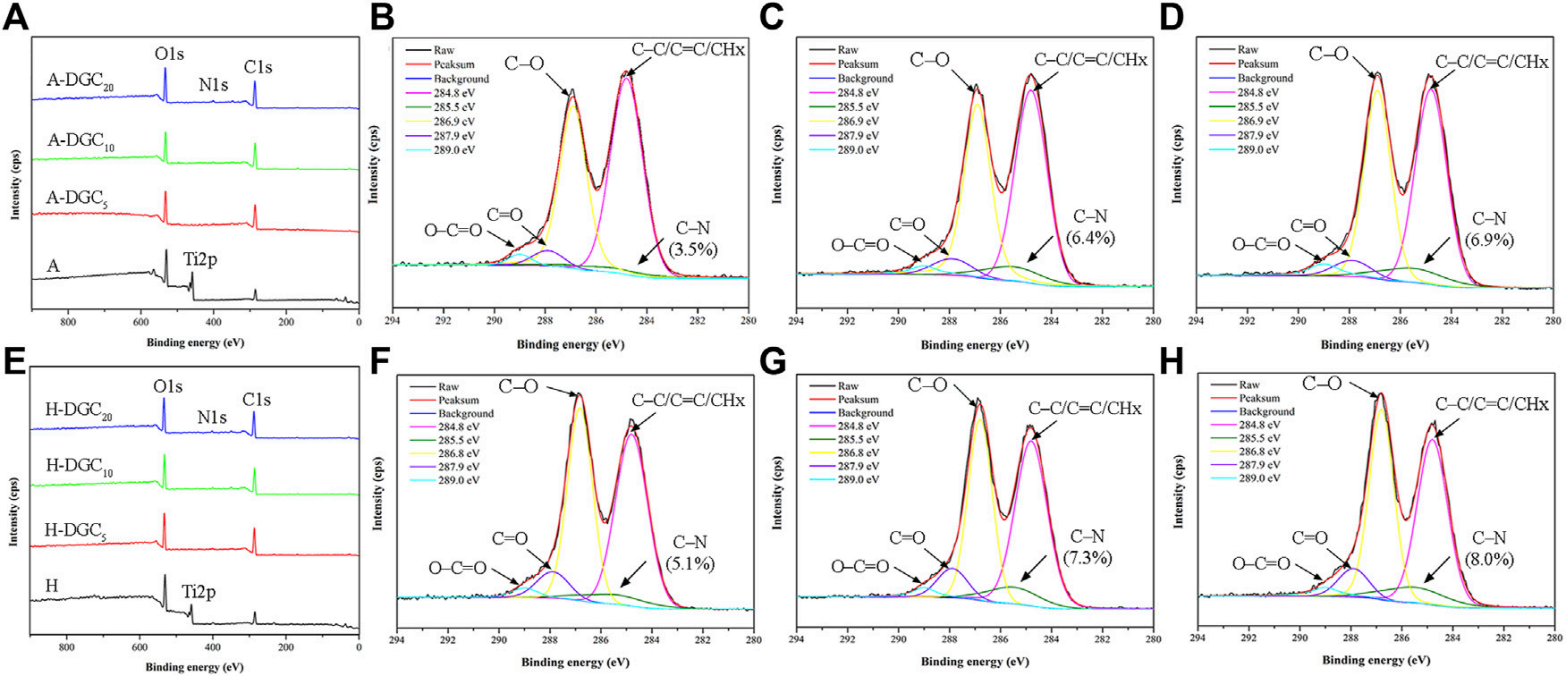
XPS results of samples. **(A)** XPS spectra of the A, A-DGC_5_, A-DGC_10_, and A-DGC_20_. High-resolution XPS spectra of C1s for the **(B)** A-DGC_5_, **(C)** A-DGC_10_, and **(D)** A-DGC_20_. **(E)** XPS spectra of the H, H-DGC_5_, H-DGC_10_, and H-DGC_20_. High-resolution XPS spectra of C1s for the **(F)** H-DGC_5_, **(G)** H-DGC_10_, and **(H)** H-DGC_20_.

The XPS spectra of the A groups and H groups are shown in Figure 4. With the coating of DGC nanofilms, the Ti2p signal disappears, whereas the N1s signal derived from the DA and COL-Ⅳ appears along with enhanced C1s signal in the DGC_n_ groups compared to the A or H group (Figures 4A,E). The XPS spectra of the A, A-DGC_5_, A-DGC_10_, and A-DGC_20_ groups reveal element signals that closely resemble those observed in the corresponding H groups. The high-resolution C1s spectra of the DGC_n_ groups were resolved into five peaks assigned to C-C/C=C/CH_x_, C-N, C-O, C=O, and O-C=O, respectively (Figures 4B–D and Figure 4F–H). The percentage of C-N in the H-DGC_5_ group (5.1%) is lower than that in the H-DGC_10_ (7.3%) and H-DGC_20_ (8.0%). However, it is worth noting that the percentages of C-N in the A groups are comparatively lower than those in the corresponding H groups.

### 3.2 Evaluation of COL-Ⅳ encapsulation

**FIGURE 5:**
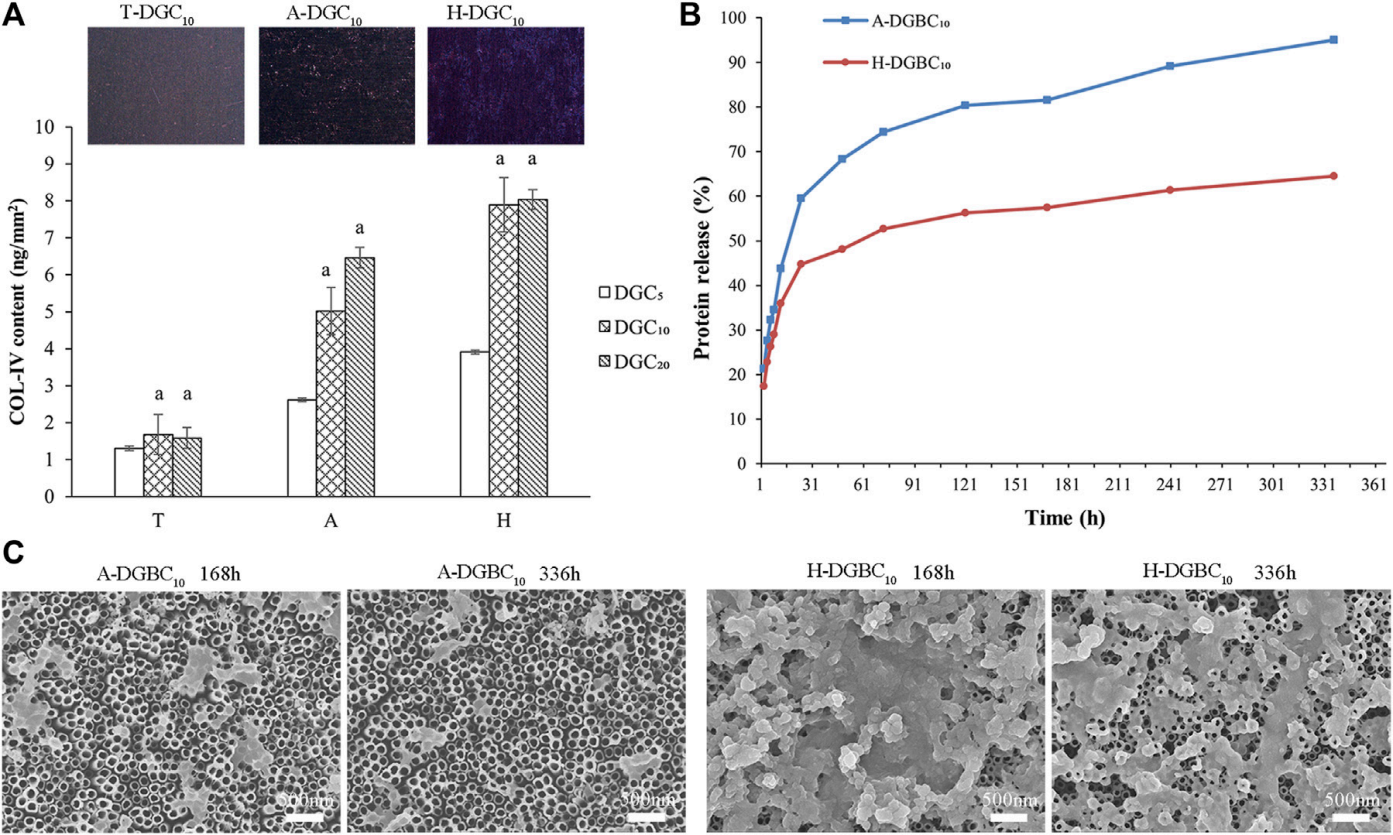
Protein content and protein release of samples. **(A)** COL-Ⅳ encapsulation in different groups. a, *p* < 0.05 vs the COL-Ⅳ content of the DGC_5_ group. The inserts show the surface COL-Ⅳ staining of the T-DGC_10_, A-DGC_10_, and H-DGC_10_. **(B)** Accumulated protein release behavior of the A-DGBC_10_ and H-DGBC_10_ surfaces after incubation in PBS at 37°C for 336 h. **(C)** Surface topographies of the A-DGBC_10_ and H-DGBC_10_ after 168 and 336 h of protein release by SEM (×30,000).

Quantitative results of the COL-IV encapsulation in the T-DGC_n_, A-DGC_n_, and H-DGC_n_ are shown in Figure 5A. The COL-Ⅳ contents in DGC_10_ and DGC_20_ are similar to each other irrespective of the group (*p* > 0.05). When loading the same number of DGC layers, the COL-Ⅳ content in the T group is the least, followed by the A group, with the H group exhibiting the most COL-Ⅳ content. The inserts in Figure 5A show the surface COL-Ⅳ staining of the T-DGC_10_, A-DGC_10_, and H-DGC_10_, with the COL-Ⅳ being distributed more evenly and abundantly on the H-DGC_10_ surface.

### 3.3 Evaluation of protein release

It can be difficult to measure the protein release in the T-DGC_10_ group because of the small amount of protein loading. Therefore, only the protein release profile of A-DGBC_10_ and H-DGBC_10_ are shown in Figure 5B. During the initial 24 h, proteins are rapidly released in both groups. In particular, the accumulated protein release in the A-DGBC_10_ group is up to approximately 60%, whereas it is at 45% in the H-DGBC_10_ group. Importantly, the protein release in the A-DGBC_10_ and H-DGBC_10_ groups is relatively slow and sustained until day 14, especially in the H-DGBC_10_ group. Within 336 h, the accumulated protein release from the A-DGBC_10_ and H-DGBC_10_ groups was 95% and 64%, respectively. The surface topographies of the A-DGBC_10_ and H-DGBC_10_ groups after 168 and 336 h of protein release are shown in Figure 5C. With the protein release, the nanofilms on the surfaces of the A-DGBC_10_ and H-DGBC_10_ groups disintegrate into nanoparticles. Moreover, the remaining DGBC components on the H-DGBC_10_ surfaces are greater than those on the A-DGBC_10_ surfaces. Some nanofilm structures remained on the surface of H-DGBC_10_ even after 336 h.

### 3.4 Evaluation of gene delivery

**FIGURE 6:**
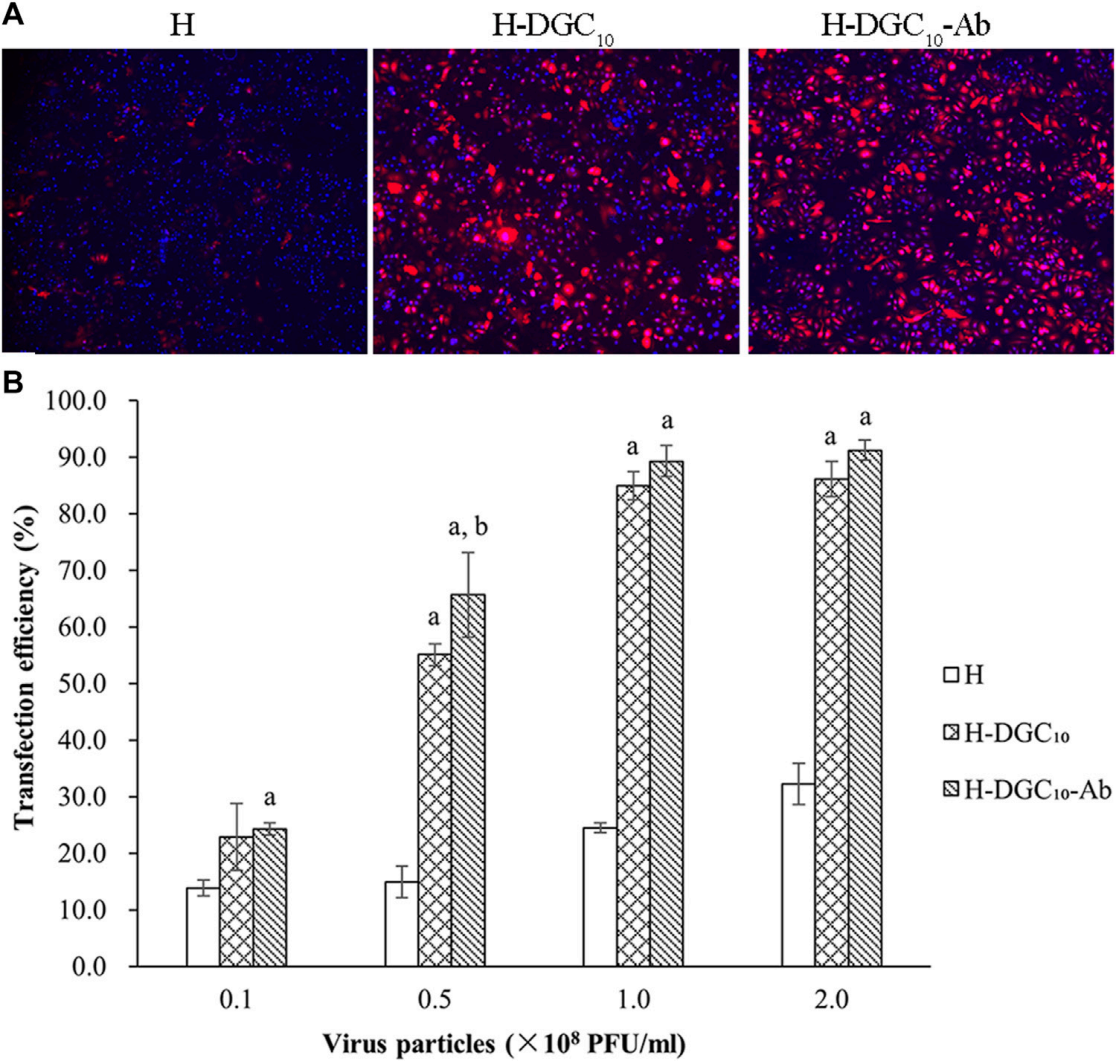
Transfection efficiency. **(A)** Representative fluorescent images of mCherry expression of cells on the H, H-DGC_10_, and H-DGC-Ab at a titer of 1.0 × 10^8^ PFU/mL. Original magnification: ×4. **(B)** Transfection efficiency in the HOECs cultured on samples at different titers. a, *p* < 0.05 vs the transfection efficiency of the H group; b, *p* < 0.05 vs the transfection efficiency of the H-DGC_10_.

The transfection efficiency of the human oral epithelial cells (HOECs) was used to evaluate the capability of different groups as gene delivery systems. Representative fluorescence images of mCherry expression on different surfaces are shown in Figure 6A. Quantitative analysis showed that transfection efficiency in the HOECs was low in all groups at a titer of 0.1 × 10^8^ PFU/mL (Figure 6B). Modification of the H surface with DGC nanofilms significantly increased the transfection efficiency of the HOECs when the titer of Ad-mCherry was no less than 0.5 × 10^8^ PFU/mL (*p* < 0.05). The transfection efficiency of the HOECs on the H-DGC_10_ and H-DGC_10_-Ab was above 85% at titers of 1.0 and 2.0 × 10^8^ PFU/mL without significant differences (*p* > 0.05).

### 3.5 Evaluation of biological effects on HOECs

#### 3.5.1 Cell viability and proliferation

**FIGURE 7:**
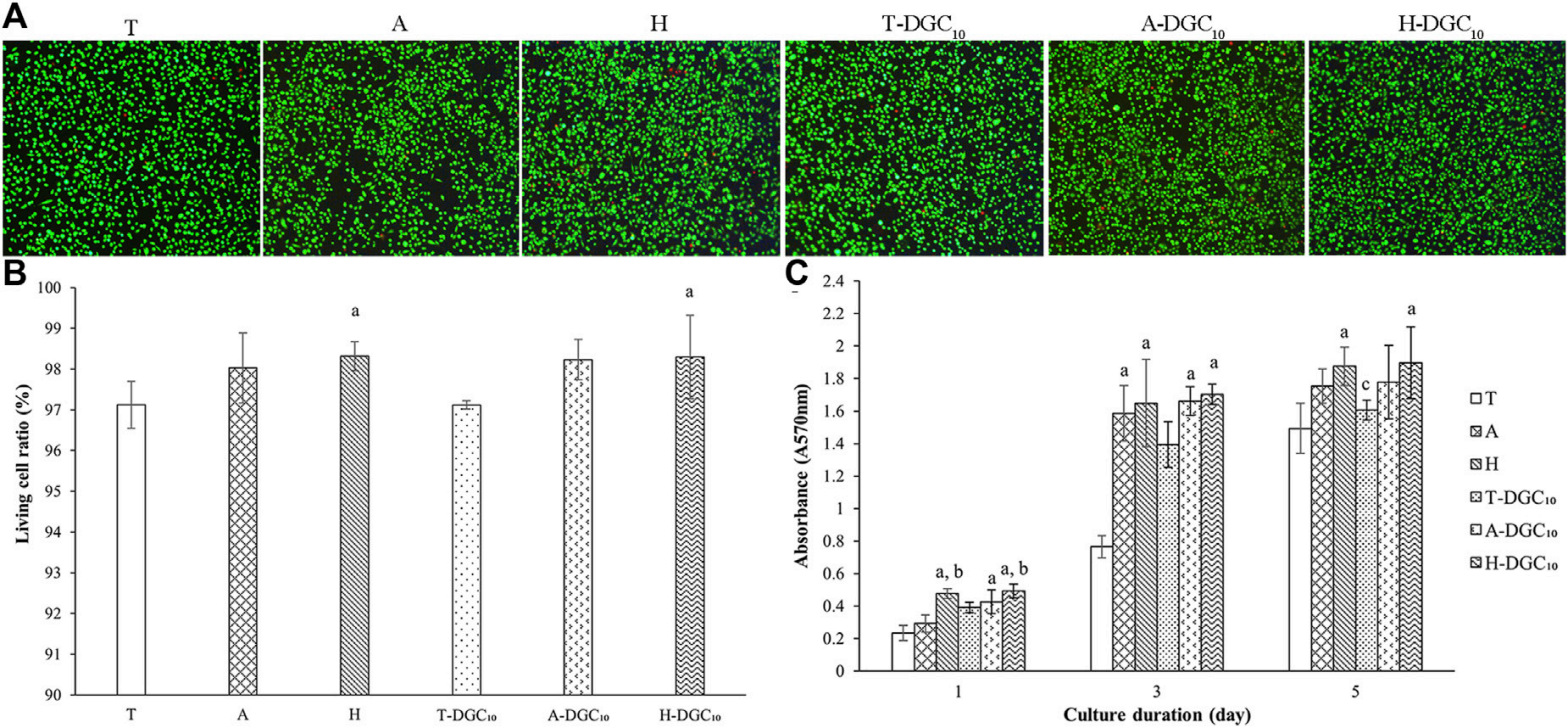
Cell viability and proliferation. **(A)** Representative live/dead staining images of the HOECs cultured on the surface of the T, A, H, T-DGC_10_, A-DGC_10_, and H-DGC_10_ for 24 h. **(B)** Living cell ratio of different samples determined from live/dead staining. a, *p* < 0.05 vs the living cell ratio of the T group. **(C)** Cell proliferation on different surfaces after culturing for 1, 3, and 5 days a, *p* < 0.05 vs the cell proliferation of the T group; b, *p* < 0.05 vs the cell proliferation of the A group; c, *p* < 0.05 vs the cell proliferation of the H group.

Representative live/dead cell-staining images are shown in Figure 7A. It is evident that there are few dead cells in all groups, indicating the excellent cytocompatibility of all surfaces. The quantitative results showed that the living cell ratio of all groups was above 97% (Figure 7B). As shown in Figure 7C, the DGC nanofilms on the T, A, and H surfaces slightly increases cell proliferation, although the difference is not statistically significant (*p* > 0.05). Cell proliferation significantly improves in the H and H-DGC_10_ groups compared to the T group (*p* < 0.05).

#### 3.5.2 Immunofluorescence

**FIGURE 8:**
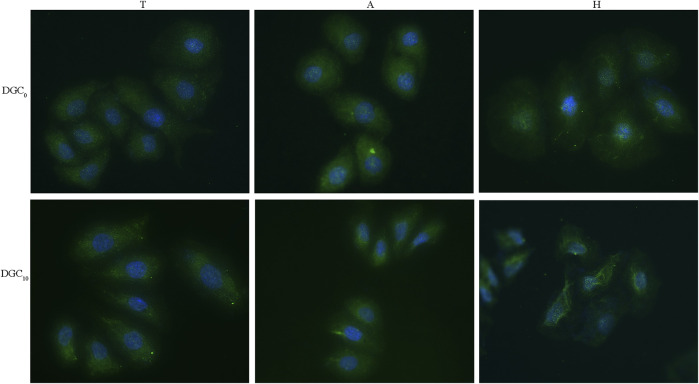
Immunofluorescence staining of integrin β4 in the HOECs cultured on different surfaces for 2 days. Original magnification: ×40. Green, integrin β4; blue, nuclei.

The expression of integrin β4 in the HOECs cultured on different surfaces is displayed in Figure 8. It is evident that integrin β4 is diffusedly distributed around the nuclei of the HOECs on the T and A surfaces, while clustered integrin β4 immunoreactivity is evident at the periphery of the HOECs on the H and DGC_10_ surfaces, especially on the H-DGC_10_ surface.

#### 3.5.3 RT-PCR

**FIGURE 9:**
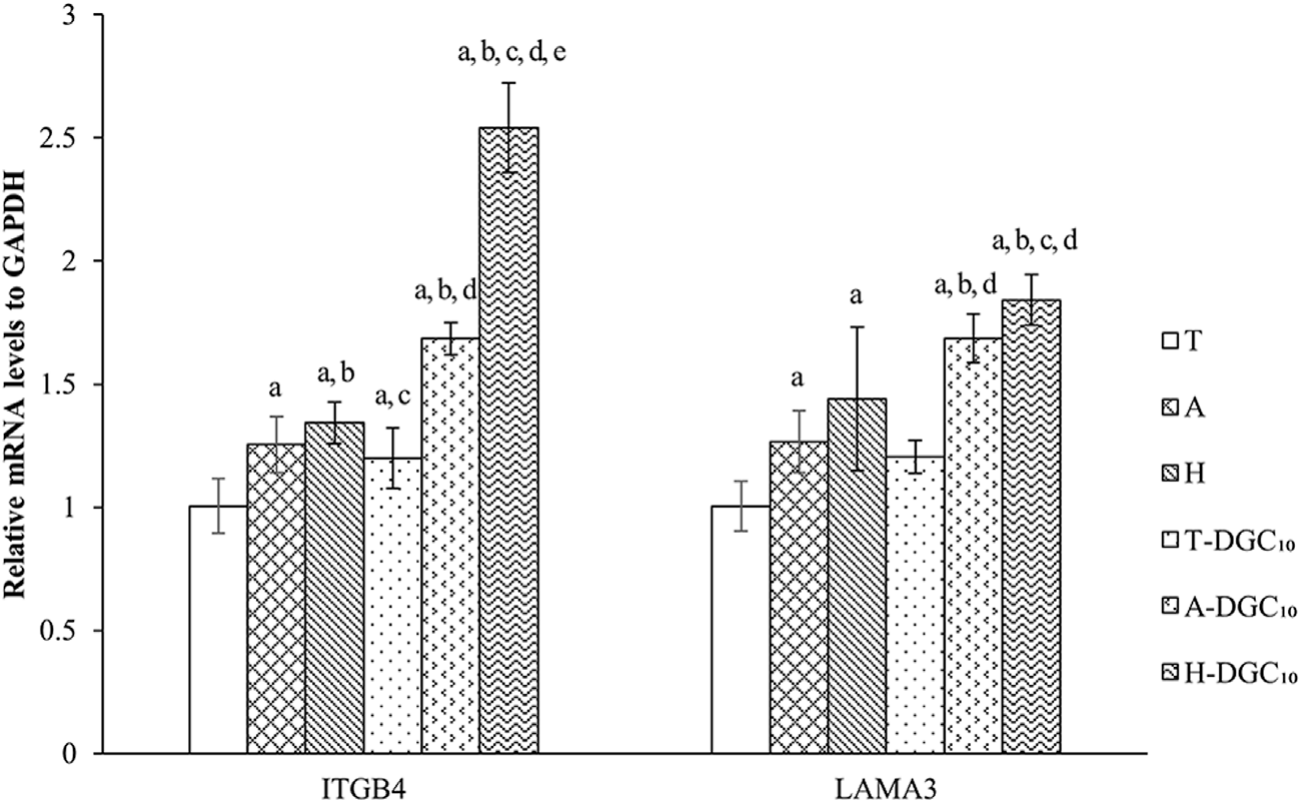
RT-PCR analysis of gene expression of HOECs cultured on the specimens for 48 h. a, *p <* 0.05 vs the relative gene expression of the T group; b, *p <* 0.05 vs the relative gene expression of the A group; c, *p <* 0.05 vs the relative gene expression of H group; d, *p <* 0.05 vs the relative gene expression of T-DGC_10_ group; e, *p <* 0.05 vs the relative gene expression of A-DGC_10_ group.

As illustrated in Figure 9, HOECs on A-DGC_10_ and H-DGC_10_ exhibit significantly higher expression levels of ITGB4 and LAMA3 when compared to the initial uncoated platforms (*p* < 0.05). Additionally, H-DGC_10_ exhibites the highest ITGB4 expression among all other groups (*p* < 0.05), corroborating the immunofluorescence results.

## 4 Discussion

This study investigated the multilayer dopamine/graphene oxide/type Ⅳ collagen (DA/GO/COL-Ⅳ, DGC) nanofilms coated on different titanium surfaces and demonstrated that ten layers of DGC nanofilms on hydrogenated TiO_2_ nanotubes significantly optimized the bioactivity and loading/delivery capability of biofunctional substances (such as proteins and gene vectors), indicating that the proposed system could be a promising template for multifunctional applications in the clinical implant field.

To choose the optimal initial platform, this study evaluated three different titanium surfaces for the DGC_n_ coating. Our results demonstrate that the DGC nanofilms on the hydrogenated titanium dioxide nanotubes (TNTs, H-DGC_n_) displayed the most uniform and stable coating surface with the best loading capacity, followed by those on the A-DGC_n_ and T-DGC_n_. A larger specific surface area was evident on the nanotubular surface of the H and A groups, providing more binding sites (Mendonca et al., 2008; Park et al., 2021) for DGC nanofilms. However, hydrogenated TNTs may generate more functional hydroxyl groups to react with DA, GO, and COL-IV. Previous studies have reported that the lattice defects (oxygen vacancies) present in hydrogenated TiO_2_ lead to a stable form of numerous hydroxyl groups on the surface (Chen et al., 2011; Lu et al., 2018), suggesting a much greater affinity for subsequent component binding. We assumed that the physicochemical characteristics of the DGC nanofilms were dependent on the specific surface area and density of the hydroxyl groups in the initial titanium platform.

We speculated that three types of components (DA, GO, and COL-Ⅳ) interlace on the substrate surface through covalent bonds, H-bonding, and electrostatic interaction to form DGC nanofilms during the layer-by-layer (LBL) process. First, the hydroxyl groups on different titanium surfaces interact with active catechol groups from the PDA (self-polymerization of DA under alkaline conditions) to form strong covalent attachments (Huang et al., 2016; Liu et al., 2016). More hydroxyl groups on the H surfaces may contribute to more covalent attachment, leading to an increased bonding strength of the H-DGC nanofilms (Figure 3C). Additionally, some of the hydroxyl groups may form hydrogen bonds with the amino, epoxy, and carboxyl groups from the PDA, GO, and COL-Ⅳ. Moreover, deprotonated hydroxyl groups could electrostatically interact with the protonated amino groups (-NH_3_^+^) of the PDA and COL-Ⅳ.

In addition to the interaction between the components and initial platforms, the interaction between each component in the LBL assembly process is crucial for DGC_n_ formation and loading capacity. The oxidated catechol groups of the PDA could form covalent bonds (quinone/semiquinone) with the amine groups of COL-Ⅳ via similar Michael addition and Schiff reactions (Lee et al., 2007; Cai et al., 2019). Subsequently, intermolecular crosslinking reactions between the PDA polymers are triggered. Our high-resolution C1s spectra showed that the percentages of C-N in the DGC_5_ groups were less than those in the DGC_10_ and DGC_20_ groups. Importantly, the H-DGC_n_ groups displayed higher percentages of C-N compared to the A-DGC_n_ groups. This difference could potentially be attributed to the initial interactive coating on H surfaces, which forms active multilayers for more component interactions. Moreover, the functional hydroxyl and carboxyl groups of GO can form ester or hydrogen bonds (Tiwari et al., 2020) with PDA and COL-IV. The inherent negative surface charge of GO (Li et al., 2008) can interact with the COL-Ⅳ, which has a positive charge in acid solution. All these interactions among the platform, PDA, GO, and COL-Ⅳ determine the composition and structure of the DGC nanofilms. Additionally, the high specific surface area of the TNTs and GO may provide more binding sites for the DGC coating and bioactive agent loading.

Our SEM results indicated instability in the nanofilms of A-DGC_20_ and H-DGC_20_, which displayed uneven and porous defects. Additionally, the Ra values and contact angles of DGC_20_ were comparable to those of DGC_10_. Our high-resolution C1s spectra demonstrated that the percentages of C-N are similar in the DGC_10_ and DGC_20_ groups. Moreover, there was no significant difference in COL-Ⅳ contents between the DGC_10_ and DGC_20_ groups (*p* > 0.05). Based on these findings, we hypothesized that the interactions among the platforms, PDA, GO, and COL-Ⅳ might be approaching saturation once the DGC nanofilms was up to 10 layers. Therefore, we have chosen to focus our further investigations, including nanofilm thickness, protein release profiles, gene delivery, and biological evaluations, exclusively on DGC_10_.

To introduce bioactive agents, peptides, proteins, drugs, or therapeutic genes should be flexibly mobilized onto the titanium surface to achieve sustainable release. In this study, the BSA and adenoviral vectors were chosen as the two model bioactive agents to evaluate the delivery and sustained-release capability of the H-DGC_10_. BSA is a standard model protein for evaluating protein adhesion and drug delivery owing to its high availability and well-understood properties. Our results demonstrate that the sustained-release ability of the H-DGC_10_ was better than that of A-DGC_10_, suggesting excellent reactivity of the H-DGC_10_ for delivery applications. We hypothesized that the H-DGC_10_ possessed more residual hydroxyl, catechol, amino, or carboxyl groups to produce a large number of reactive sites and electrostatic interactions for substance loading and sustained release. The H group, characterized by its superhydrophilic surface and abundance of hydroxyl groups, might lead to a more interactive initial DA/GO/COL-Ⅳ layers on surfaces. These layers subsequently created additional binding sites for loading and controlled release of other components. As a result, H-DGC_10_ nanofilms achieved a superior sustained-release profile compared to A-DGC_10_. While DA and GO were incorporated into our multilayer nanofilms owing to their universal adhesive property suitable for numerous organic/inorganic surfaces, further investigations are required to understand the specific interactions between the nanofilms and different substances at different concentrations.

The timely establishment of peri-implant soft-tissue integration requires 7 days for initial epithelial attachment, and another 2–8 weeks for epithelial and connective tissue maturation (Hämmerle et al., 2014; Guo et al., 2021). Consequently, the release of bioactive agents during soft-tissue integration is crucial. Our results indicate that the drug release of the H-DGC_10_ group was sustained for 2 weeks, still retaining 40% unreleased; this supported the initial attachment of peri-implant soft tissue. In other words, the release profile of the H-DGC_10_ conforms to the soft-tissue healing process.

Viral vectors are widely used in gene therapy owing to their high transfection efficiency (Lee et al., 2017). However, unlike negatively charged nucleic acids, viral vectors can be difficult to immobilize on titanium surfaces for *in situ* gene delivery. Substrate-mediated gene delivery—a method to immobilize therapeutic genes directly on the substrate—has been reported to improve transfection efficiency by increasing the local gene concentration, leading to reduced gene doses and favorable biosecurity (Mantz et al., 2019; Laird et al., 2020; Puhl et al., 2023). However, therapeutic genes were more or less exposed to the process of loading or modification in most studies. Zhang *et al.* (Zhang et al., 2018) functionalized a titanium-coated surface with anti-adenovirus antibodies to immobilize adenovirus vectors. However, this functionalization method proved to be too limited for widespread application in other vectors. This study designed a more flexible coating for *in situ* gene delivery, and the H-DGC_10_ displayed excellent transduction efficiency of up to 90% with a low dose of vectors, thereby improving the biosafety. We inferred that the PDA and GO layers on H-DGC_10_ provided plenty of reactive sites for immobilizing gene vectors.

The junctional epithelium, which seals the peri-implant soft tissue, attaches to the transmucosal implant surfaces through cell-matrix structures comprising hemidesmosomes (HDs) and the basement membrane (BM) (Gould et al., 1984; Atsuta et al., 2019). Our results show that the thickness of the DGC nanofilms on the A-DGC_10_ and H-DGC_10_ was around 200 nm, which is similar to the bond width of the BM (60 nm–150 nm) (Stern, 1981; Atsuta et al., 2016). We hypothesized that DGC nanofilms play biomimetic and early adhesive roles in the initial epithelial sealing stage. When the DGC nanofilms disintegrates (Figure 5C), the cell-material interface is gradually constructed with a natural matrix secreted by the epithelial cells.

Our *in vitro* investigations show that the DGC nanofilms grown on different substrates exhibited good biocompatibility with the HOECs. Laminin 332, a pivotal BM protein, has been reported to interact with integrin α6β4 to modulate cell adhesion and HD formation (Larjava et al., 2011; Taniguchi et al., 2020; Te Molder et al., 2021). Mirjam *et al.* (Nievers et al., 2000) verified that integrin β4 could bind to HD1/plectin to form HD-like structures. In this study, the H-DGC_10_ significantly promoted the clustering of integrin β4 and the gene expression levels of integrin β4 and laminin 332, which could participate in HD formation. Additionally, the plentiful binding sites on the H-DGC_10_ surfaces could improve the adhesion of the HOECs, and the COL-Ⅳ molecules could interact with membrane receptors to facilitate the secretion of functional matrixes for the BM assembly and cell anchorage (Khoshnoodi et al., 2008; Takeuchi et al., 2010). We speculated that the superior performance of H-DGC_10_ could be attributed to the larger amount of active binding sites facilitating initial ECM protein adsorption, as well as a greater encapsulation of COL-Ⅳ with bioinspired properties when compared to other groups.

In this study, we investigated the characteristics of DGC nanofilms on different titanium surfaces, their potential application as *in situ* carrying, sustained releasing, and delivering agents, and their effects on epithelial cells. However, this study had a few limitations with respect to the detailed analysis of the interactions in the LBL process, comprehensive evaluation of the H-DGC_n_ as a versatile carrier, and its unique biological properties. Therefore, follow-up studies should be conducted to thoroughly evaluate the effects of the H-DGC_n_ on the controlled release profile *in vitro* and *in vivo*, as well as its biological effectiveness, including its immunomodulatory and antibacterial properties. Despite the limitations of this study, it remains particularly valuable for the long-term success of titanium implants, where versatile therapeutic strategies could be required in emergencies.

## 5 Conclusion

In this study, multilayer DGC nanofilms were fabricated on different Ti surfaces using the LBL technique. The hydrogenated TNTs proved to be the best platform for coating the DGC nanofilms, which achieved stability after the number of nanofilm layers increased to 10. The H-DGC_10_ displayed excellent loading capacity, sustained-release capability, and *in situ* gene delivery abilities. *In vitro* investigations demonstrated the biocompatibility of the H-DGC_10_ in epithelial cells. Consequently, a versatile and bioactive DGC multilayer coating could be developed on hydrogenated TNTs for expanded clinical applications.

## Data Availability

The original contributions presented in the study are included in the article/Supplementary Material, further inquiries can be directed to the corresponding author.
